# The Effect of Lag Screw Position on Rotational Stability and Stress Concentration in Unstable Basicervical Intertrochanteric Fractures: A Finite Element Analysis

**DOI:** 10.3390/jcm15114375

**Published:** 2026-06-05

**Authors:** Se-Won Lee, Min-Seok Kim, Sung-Jae Lee, Dae-Kyung Kwak, Je-Hyun Yoo

**Affiliations:** 1 Department of Orthopedic Surgery, Yeouido St. Mary’s Hospital, College of Medicine, The Catholic University of Korea, Seoul 07345, Republic of Korea; ssewon@naver.com; 2 Department of Biomedical Engineering, Inje University, Gimhae 50834, Republic of Korea; kms20162296@gmail.com (M.-S.K.); biomech100@gmail.com (S.-J.L.); 3 Department of Orthopedic Surgery, Bundang Jesaeng General Hospital, Seongnam 13590, Republic of Korea; limitkdk@gmail.com; 4 Department of Orthopedic Surgery, Hallym University Sacred Heart Hospital, College of Medicine, Hallym University, Anyang 14068, Republic of Korea

**Keywords:** hip, unstable intertrochanteric fracture, basicervical fracture type, cephalomedullary nailing, lag screw, finite element analysis

## Abstract

**Background/Objectives**: Due to the inherent rotational instability of the proximal fragment in unstable basicervical intertrochanteric (IT) fractures, the biomechanical effect of lag screw position may differ from that observed in typical unstable IT fractures. This study aimed to evaluate the influence of lag screw positioning on proximal fragment displacement and stress distribution after cephalomedullary nailing (CMN) in unstable basicervical IT fractures using finite element analysis. **Methods**: Twelve finite element models of unstable basicervical IT fractures fixed with a CM nail were constructed with lag screws placed in four anteroposterior (AP) positions (superior 5 mm, center, inferior 5 mm, and inferior 10 mm) and three axial positions (anterior, center, and posterior). The positional change of the proximal fragment and stress concentration on the nail construct were measured. **Results**: In this computational model, proximal fragment displacement and stress concentration, including peak von Mises stress and mean stress over a region of interest, increased as the lag screw was positioned more inferiorly on the AP view and more posteriorly on the axial view. Conversely, a relatively superior-anterior lag screw position was associated with the lowest proximal fragment displacement and reduced stress concentration on the nail construct and around the lag screw tip. **Conclusions**: Within the limitations of this finite element analysis using a single femoral model and axial loading condition, a relatively superior-anterior lag screw position was associated with more favorable biomechanical behavior compared with more inferior or posterior positions. These findings should be interpreted as hypothesis-generating biomechanical observations rather than direct clinical guidance.

## 1. Introduction

Basicervical intertrochanteric (IT) fractures are a specific type of pertrochanteric fracture in which the main fracture line crosses the base of the femoral neck at its junction with the IT region [1,2]. In this study, an unstable basicervical IT fracture was defined as a fracture with the main fracture line located at the base of the femoral neck, combined with unstable pertrochanteric features corresponding to AO/OTA 31-A2 morphology, including posteromedial comminution and greater trochanteric fragmentation [3]. Due to the narrow base of the proximal fragment and reduced contact area at the fracture site, these fractures are likely to have inherent rotational instability of the proximal fragment compared to typical IT fractures. Moreover, in unstable basicervical fracture types accompanied by trochanteric comminution and/or a large posteromedial fragment, the risk of fixation failure due to the rotational instability of the proximal fragment after osteosynthesis may increase even more [4,5]. Su et al. [2] reported that basicervical fracture patterns may be more prone to collapse and rotational instability than typical IT fractures. Ciufo et al. [6] reported that a basicervical fracture component and lateral wall fracture along with trochanteric comminution are risk factors associated with cut-out after cephalomedullary nailing (CMN) in trochanteric hip fractures.

CMN is widely performed as a surgical treatment of most pertrochanteric fractures because it is less invasive and biomechanically superior to extramedullary devices [7]. Generally, the quality of reduction and the location of the lag screw within the femoral head have been known to have significant impacts on the surgical outcomes, especially in unstable IT fractures. To date, for conventional IT fractures, the optimal lag screw position is widely considered to be the center-to-inferior position on the anteroposterior (AP) view and center on the axial view to minimize fixation failure [8,9,10]. However, we have often experienced fixation failures even under recommended anatomical or good reduction and lag screw position after CMN in unstable IT fractures with a basicervical fracture component. Therefore, we suspected that the optimal lag screw position in unstable basicervical IT fractures may differ from the optimal one in conventional unstable IT fractures due to the narrower, more rotationally unstable proximal fragment. However, despite its clinical importance, the optimal lag screw position for this specific fracture subtype has not yet been addressed in the biomechanical and clinical literature.

Given the more rotationally unstable nature of the proximal fragment in these specific fractures, we hypothesized that lag screw position may influence proximal fragment displacement and stress distribution differently from conventional unstable IT fractures. Therefore, the aim of this finite element analysis study was to compare the biomechanical effects of different lag screw positions on proximal fragment stability and stress distribution after CMN in unstable basicervical IT fractures.

## 2. Materials and Methods

### 2.1. Finite Element Model (FEM)

**Figure 1:**
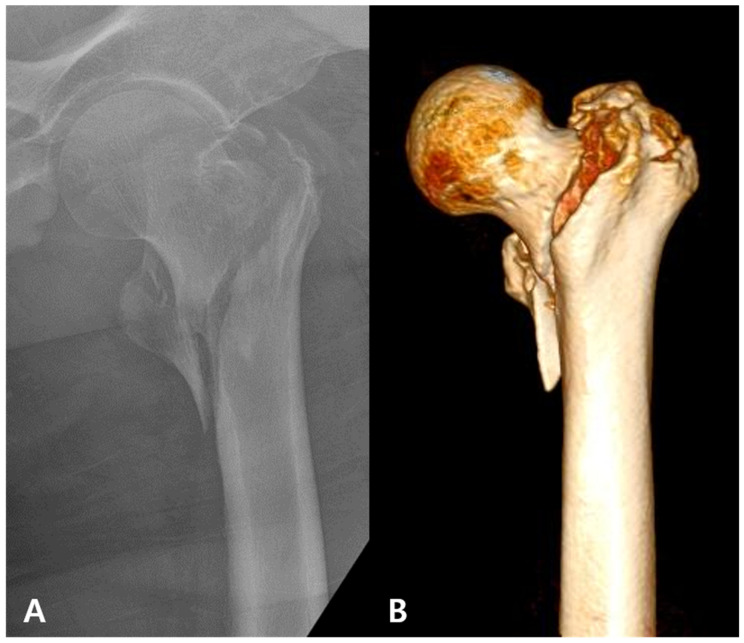
Anteroposterior radiograph (**A**) and 3-D CT image (**B**) showing unstable basicervical IT fracture of the left proximal femur.

A previously verified three-dimensional (3-D) FEM was used for this study [11,12]. For an intact left femur, computed tomography (CT) was used, with 1.0 mm resolution incremented every 1.0 mm. Using CT slice images produced by Mimics application version 21.0 (Materialise, Leuven, Belgium), the geometry of the femur was reproduced. This geometry was used to produce the cortical and cancellous bone volume, and an FEM was implemented through the ABAQUS program (Dassault Systems, Paris, France). Geometry extraction was used to create an intact femoral shape, which was then volume meshed with isotropic tetrahedral elements. Strain was measured to validate the FEM and compared with that of a previous study by connecting a strain gauge at 20 sites on the anterior, posterior, medial, and lateral sides of the FEM [12]. A previously verified model was used to replicate an osteoporotic bone model [13]. To replicate the unstable IT fracture, the automatic solid and mesh generation program (ABAQUS) was used. To simulate the basicervical fracture pattern, the main fracture line was created close to the base of the femoral neck, and the lesser trochanter and part of the greater trochanter including its tip were consistently removed to assume the clinical worst case of their comminution (Figure 1). In this study, an unstable basicervical IT fracture was defined as a fracture with the main fracture line located at the base of the femoral neck, combined with unstable pertrochanteric features corresponding to AO/OTA 31-A2 morphology, including posteromedial comminution and greater trochanteric fragmentation [3]. The fracture gap was set at 3 mm to meaningfully compare the numerical results of femoral head rotation and other biomechanical changes according to physiological load application to finite element models.

Short Gamma 3 nails (Stryker, Mahwah, NJ, USA) with a length of 170 mm, diameter of 12.0 mm, caput–collum–diaphyseal angle of 125°, lag screw length of 100 mm, and distal locking screw length of 28 mm were used. Based on this design, an implant 3-D model was produced to meet the femur specifications used in this study. The lag screw was positioned at 5 mm intervals on the AP view in the femoral head and 3 mm intervals on the axial view, and the tip apex distance (TAD) was maintained below 25 mm in all models based on the initial implant configuration. The finite element simulations were performed with a standardized 3 mm fracture gap to compare biomechanical behavior among lag screw positions under identical loading and boundary conditions.

**Figure 2:**
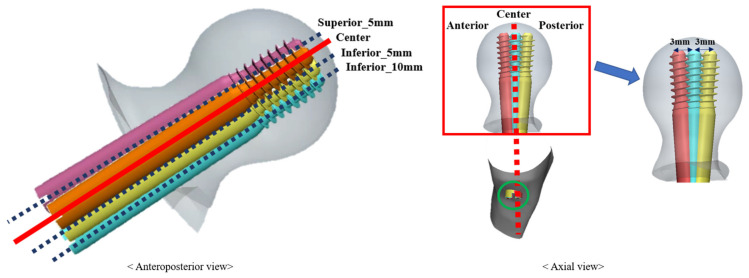
Finite element models with different lag screw positions on anteroposterior view and axial view.

The positional intervals of 5 mm on the AP view and 3 mm on the axial view were determined through iterative preliminary testing. When the lag screw was positioned too eccentrically within the femoral head, the effective screw length within the bone was shortened and the screw tip was located in a region with relatively poor bone stock, which may reduce the screw’s load-bearing capacity and increase the risk of fixation failure. Conversely, when the intervals were set below the selected values or positioned near the center of the femoral head, no meaningful biomechanical differences were observed between models. Twelve models were implemented using a combination of four different positions on the AP view and three different positions on the axial view (Type 1: superior 5 mm and anterior 3 mm; Type 2: superior 5 mm and center; Type 3: superior 5 mm and posterior 3 mm; Type 4: center and anterior 3 mm; Type 5: center and center; Type 6: center and posterior 3 mm; Type 7: inferior 5 mm and anterior 3 mm; Type 8: inferior 5 mm and center; Type 9: inferior 5 mm and posterior 3 mm; Type 10: inferior 10 mm and anterior 3 mm; Type 11: inferior 10 mm and center; Type 12: inferior 10 mm and posterior 3 mm) (Figure 2).

The mesh of the Gamma 3 nail and fractured femur model was constructed using ABAQUS utilizing eight-node hexahedral and four-node tetrahedral elements. These elements sustained contact conditions in the fracture plane and allowed for the definition of different material properties.

### 2.2. Material Properties

In this study, the bone model was assumed to have homogeneous, isotropic, and linear elastic properties. For cancellous bone, the elastic modulus (E) was determined using a previously reported mean CT Hounsfield unit (HU) value of 120.8 ± 41.8 for the femur [14]. The relationship among HU, apparent density (ρ), and elastic modulus was defined as follows:

ρ = 0.131 + 0.001067 HU

E = 6850ρ^1.49^

where ρ is the apparent density (g/cm^3^) and E is expressed in Mpa [15].

The material properties of the bone and nail were cited from previous studies (Table 1) [16,17]. Titanium alloy (TI6Al4V) was used for the Gamma 3 nail (Young’s modulus, 114 GPa; Poisson’s ratio, 0.34) for the purpose of analysis. The material properties of cortical bone and cancellous bone were applied to the same femoral regions.

### 2.3. Boundary and Loading Conditions

**Figure 3:**
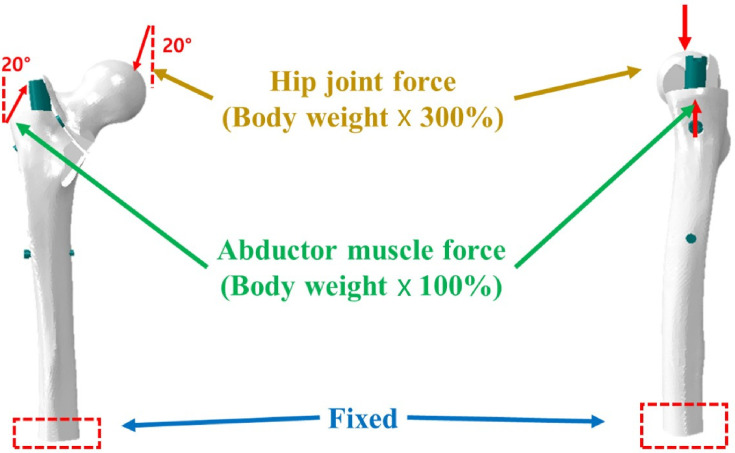
Loading condition of the analysis model; hip joint force, 2013.9 N (body weight × 300%); abductor muscle force, 671.3 N (body weight × 100%).

Assuming the one-leg stance, the hip joint reaction force on the femoral head and abductor muscle force on the lateral surface of the greater trochanter were applied at an angle of 20° from the vertical line in the frontal plane (Figure 3) [18,19]. There are two contact (‘tied’ and ‘general’) conditions for performing the finite element analysis and both conditions were used to implement the motion caused by physiological load application. First, a tied contact condition was applied between cortical and cancellous bone to represent complete bonding, and also between the bone and lag screw and between the bone and distal locking screw. Second, to allow for ideal movement between the nail and bone, a 0.42 friction coefficient of the ‘general’ contact condition was applied. For the interactions between the proximal and distal bone fragments and the nail and lag screw, the friction coefficients were set at 0.46 and 0.23, respectively [20].

### 2.4. Finite Element Analysis (FEA)

**Figure 4:**
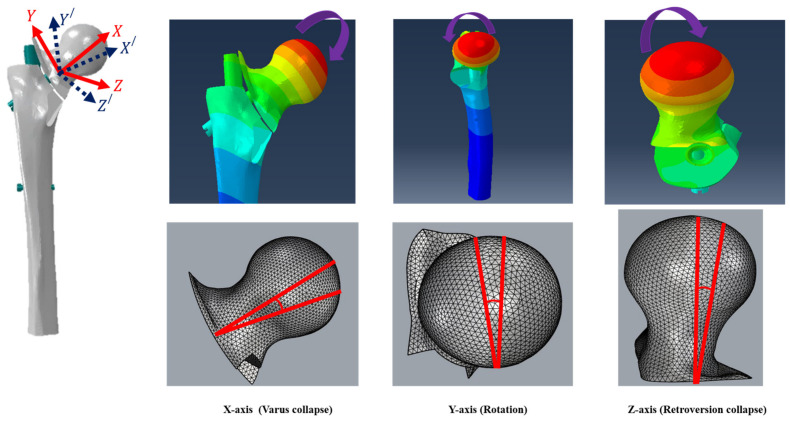
Definition of the migration direction for the assessment of 3-dimensional migration of the proximal fragment.

To investigate the positional change of the proximal fragment, element nodes were set at the superior, center, and inferior of the femoral head by referring to the Bryant angle principle, which can assess the 3-D angle difference through a 3-D coordinate system [21]. Three axes (X-, Y-, and Z-axes) were set based on those determined by the default setting in the 3-D modeling and finite element analysis program. Before and after axial loading was applied to each FEM, the 3-D position of the proximal fragment was measured according to each axis (X-axis, varus collapse; Y-axis, rotation; Z-axis, retroversion collapse) (Figure 4) [22]. Stress concentrations on the nail and within the femoral head were also examined. The mean stress over a region of interest (ROI) was measured in each FEM and compared to the yield strength. We set the regions in which the peak von Mises stresses (PVMSs) on the nail construct and within femoral head were observed as the ROIs of each FEM. The yield strength of the nail construct was referenced from a previous study (Ti6Al4V, 880 MPa) [23]. The yield strength of cancellous bone was set at 5 MPa based on reported values for osteoporotic cancellous bone [24]. Because this study was designed as a deterministic finite element analysis using a previously validated computational model, biological replicates were not applicable and technical replicates were not performed. Each of the twelve finite element models represented a unique lag screw configuration and were analyzed once under identical loading and boundary conditions. Accordingly, formal statistical analysis was not performed. The biomechanical parameters obtained from each model were compared descriptively under identical simulation conditions.

## 3. Results

### 3.1. Positional Change of the Proximal Fragment

**Figure 5:**
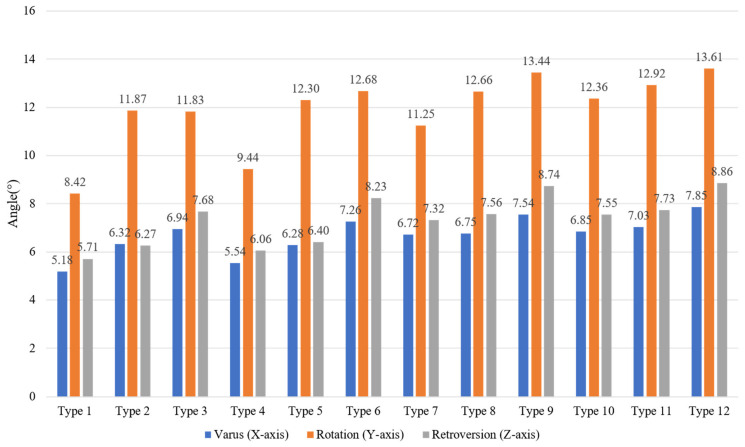
Results of the positional change of the proximal fragment.

Table 2 presents the positional change of the proximal fragment according to three axes across 12 different FEMs (Type 1 to 12) with the increase rate relative to Type 1 shown in parentheses. The positional change of the proximal fragment was minimal in Type 1 (5 mm superior on the AP view, 3 mm anterior on the axial view). The FEMs (Types 2 to 12) generally showed greater positional change of the proximal fragment compared to the baseline (Type 1). It increased as the lag screw moved from superior to inferior on the AP view and from anterior to posterior on the axial view. Consequently, the greatest positional change of the proximal fragment occurred in Type 12 (10 mm inferior on the AP view, 3 mm posterior on the axial view) (Figure 5). Meanwhile, Type 4 (center on the AP view, 3 mm anterior on the axial view) showed the most similar results to Type 1 although the former were slightly greater. Along all three axes, similar results were observed in each type. The values on the X- and Z-axes were similar or slightly greater on the Z-axis. However, the values on the Y-axis were greatest across all FEMs. In other words, the rotation of the proximal fragment was substantially greater than varus and retroversion collapses across all models.

### 3.2. Stress Distribution at the Nail Construct and Within the Femoral Head

**Figure 6:**
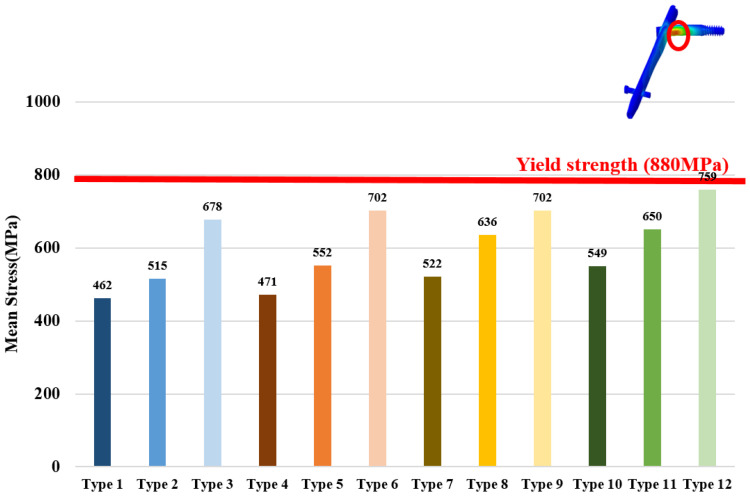
Results of the mean stress over a region of interest on the nail construct in each finite element model.

The PVMSs on the nail construct in each FEM were observed at the inferior portion of the lag screw at the medial contact area of the nail–lag screw junction. Therefore, we set this area as the ROI of the nail construct in each FEM (Figure 6). The mean stress over the ROI was lowest in Type 1 and increased as the lag screw position moved inferiorly and posteriorly. Consequently, the mean stress was greatest in Type 12, which reached 86% of the yield strength (759 MPa). The mean stresses in all FEMs and the increase rates compared to Type 1 are presented in Table 3.

Stress concentration within the femoral head showed patterns similar to those observed on the nail construct. The PVMS in each FEM was observed around the lag screw tip, which was set as the ROI within the femoral head in each FEM. The mean stress over the ROI was also lowest in Type 1, similar to stress concentration on the nail construct. Only Type 1 (superior-anterior position) maintained a mean stress below the reference yield strength of osteoporotic cancellous bone (5 MPa). It should be noted, however, that exceeding this threshold in a linear elastic model does not directly equate to clinical cut-out or fixation failure, as this model did not incorporate damage accumulation, fatigue, or progressive plastic deformation mechanisms. These findings should therefore be interpreted as relative comparative indicators of stress concentration among lag screw positions rather than as absolute predictors of clinical outcome. In addition, the mean stresses increased as the lag screw position within the femoral head moved from superior to inferior on the AP view and from anterior to posterior on the axial view. The mean stresses increased substantially as the lag screw was positioned posteriorly on the axial view and inferiorly on the AP view. Type 4 (center-to-anterior) showed the most similar value to Type 1 (superior-to-anterior) although the former was slightly greater. Table 4 presents the mean stresses and the increase rates in each FEM compared with those in Type 1 (superior-to-anterior).

## 4. Discussion

Compared to the conventional IT fractures, basicervical IT fractures have been reported to have a higher failure rate after CMN due to the greater rotational instability of the proximal fragment, which may be caused by a shorter proximal fragment and subsequently narrower contact area at the main fracture site [2,3]. Therefore, the appropriate position of the lag screw within the femoral head as well as the reduction quality are of paramount importance to reduce fixation failure after CMN in unstable IT fractures with a basicervical fracture component. Several authors have reported that a basicervical fracture component is a risk factor for fixation failure after CMN in unstable basicervical IT fractures, and more careful attention is needed when performing CMN in these fractures [25,26]. Furthermore, we have experienced several failed cases despite achieving anatomical reduction and a well-positioned lag screw (center or inferior on the AP view and center on the axial view) within a TAD < 25 mm in unstable basicervical IT fractures. Accordingly, we hypothesized that lag screw position may influence biomechanical behavior in unstable basicervical IT fractures and conducted this FEA study to compare these effects.

This FEA study revealed that the positional change of the proximal fragment and stress concentration at the nail–bone construct in unstable basicervical IT fracture models fixed with a cephalomedullary nail increased as the lag screw position moved from superior to inferior on the AP view and from anterior to posterior on the axial view. Consequently, the stress concentration at the nail–bone construct and the positional change of the proximal fragment were lowest in the superior-to-anterior position of the lag screw within the femoral head. The center-to-center and inferior-to-center position of the lag screw showed greater stress concentration and positional change of the proximal fragment than the superior-to-anterior position under the assumption of anatomical reduction. Meanwhile, the center-to-anterior position of the lag screw showed the stability and stress distribution most similar to the superior-to-anterior position although those were slightly higher in the center-to-anterior position. As a result, the present study found that these two positions demonstrated the most favorable biomechanical profile in this computational model, which differs from the conventionally recommended position in conventional IT fractures, and we believe that they may be the most resistant to the spinning motion of the proximal fragment due to its rotational instability, considering femoral anteversion. As Type 1 (superior-anterior) and Type 12 (inferior-posterior) represented the two extremes of the clinically feasible positional range evaluated in this study, their comparison was used to illustrate the maximum potential biomechanical difference achievable through lag screw repositioning. Although a universally accepted angular threshold for clinical failure in unstable basicervical IT fractures has not been established, the relative increases observed in the present study may be clinically meaningful. Compared with the superior-anterior position, the inferior-posterior position showed a 51.5% increase in varus collapse, a 61.6% increase in rotational displacement, and a 55.2% increase in retroversion collapse. In a short and inherently rotationally unstable proximal fragment, repetitive cyclic loading may amplify these initial angular changes and contribute to progressive micromotion, loss of fixation, cut-out, or implant failure.

The optimal lag screw position within the femoral head remains a cornerstone of successful CMN for IT fractures. For conventional unstable IT fractures, biomechanical and clinical studies have consistently recommended a center-to-inferior position on the anteroposterior view and a central position on the axial view to minimize the risk of cut-out and fixation failure [8,9]. The rationale is based on the higher bone density and biomechanical advantage of the central and inferior regions of the femoral head. However, the present finite element analysis demonstrated that this established paradigm may not be directly transferable to unstable basicervical IT fractures. In contrast to the center-inferior position advocated by Kane et al. [9], our results showed that the superior-to-anterior lag screw position yielded the lowest positional change of the proximal fragment and the lowest stress concentration both at the nail–lag screw junction and around the lag screw tip.

This discrepancy may be attributed to the distinct biomechanical behavior of unstable basicervical fractures. Unlike typical IT fractures, the main fracture line in basicervical fracture types exits at the base of the femoral neck, resulting in a shorter proximal fragment and a substantially reduced fracture contact area. Consequently, the proximal fragment is inherently more susceptible to rotational displacement than to varus collapse, as evidenced by the predominance of rotational movement along the Y-axis observed in all finite element models in the present study. Therefore, while the center-inferior lag screw position may be advantageous in conventional IT fractures because of stronger bone stock support, unstable basicervical fractures may require a lag screw position that more effectively resists rotational instability of the proximal fragment. In this context, the superior-to-anterior position appears to function biomechanically as an anti-rotation buttress against rotational forces generated during physiological loading.

To the best of our knowledge, the present study is the first finite element analysis specifically evaluating the biomechanical effect of lag screw position in unstable basicervical IT fractures rather than generalized IT fracture patterns. These findings suggest that implant positioning strategies in unstable basicervical fractures may need to differ from those traditionally recommended for conventional IT fractures. Therefore, the present findings should be interpreted as biomechanical behavior under the specific axial loading conditions applied in this finite element model, and they may not fully represent the complex multidirectional loading conditions encountered in clinical practice.

In terms of the stress concentration on the nail construct and around the lag screw tip, the increase rates were much higher in the posterior position on the axial view than those in the inferior position on the AP view. This indicates that the stress concentration is more affected by the lag screw position on the axial view than on the AP view. Finally, the posterior position of the lag screw on the axial view was associated with the greatest stress concentration and positional change of the proximal fragment within this computational model, and therefore demonstrated the greatest biomechanical disadvantage among the positions tested. The precise extent to which the lag screw should be positioned superiorly or anteriorly within the femoral head remains unclear. It is well known that the center of the femoral head has a higher bone mineral density and stronger holding power [27,28]. Uemura et al. [27] reported that the bone mineral density in the superior portion was slightly lower than that in the center on the AP view, but the difference was not statistically significant. Although it is challenging to determine the exact position of the lag screw to guarantee successful bony union without fixation failure in unstable basicervical IT fractures, the present findings suggest that a relatively superior-to-anterior position was associated with lower rotational displacement and stress concentration within this computational model. Therefore, based on the present computational model, the superior-to-anterior position from the center axis of the femoral head appeared to represent a biomechanically reasonable range while avoiding problems associated with excessive eccentricity of the lag screw. It must be acknowledged that superior lag screw placement has traditionally been associated with an increased risk of superior cut-out, based on the relatively lower bone mineral density of the superior femoral head [27]. While the present finite element analysis suggests that a relatively superior-anterior lag screw position may offer biomechanical advantages in terms of rotational stability within this specific computational model, this should not be interpreted as a general recommendation for superior placement in clinical practice. The trade-off between rotational stability and cut-out risk must be carefully individualized based on each patient’s femoral head geometry, bone quality, reduction quality, and TAD. Surgeons should exercise particular caution when considering a more superior lag screw position in patients with severe osteoporosis, a small femoral head, or suboptimal reduction.

Several limitations must be acknowledged. First, this study employed a single-stance axial loading condition and did not simulate torsional, cyclic, or multidirectional loads encountered during gait, stair-climbing, sit-to-stand transitions, stumbling, or other postoperative activities. The absence of torsional loading is a particularly important limitation given that the primary hypothesis of this study concerns rotational instability of the proximal fragment. Torsional forces may substantially alter the relative biomechanical behavior of different lag screw positions, and future studies incorporating torsional and cyclic loading protocols would provide a more complete biomechanical characterization. Second, only one fracture pattern with a 3 mm fracture gap was modeled; thus, the influence of varying degrees of comminution, reduction quality, and fracture morphology was not assessed. Third, the finite element model was based on CT data from a single femur and therefore did not reflect inter-individual anatomical variability. Fourth, the bone material was modeled as homogeneous, isotropic, and linearly elastic, which does not fully capture the heterogeneous and anisotropic nature of osteoporotic proximal femoral bone. This simplification may affect the stress distribution around the lag screw tip and within the femoral head, particularly given the relatively small positional differences being compared. Future studies incorporating anisotropic and heterogeneous bone material models would provide a more accurate representation of in vivo biomechanical conditions. Fifth, the tied contact condition applied at the bone–lag screw interface assumes complete osseous integration and prevents micromotion at the screw–bone interface. This assumption may not accurately reflect the interface compliance encountered in osteoporotic cancellous bone in clinical practice, and it could influence the apparent relative advantage of one lag screw position over another. Future studies incorporating frictional or cohesive contact formulations at the bone–screw interface would provide a more realistic representation of this clinically important interaction. Sixth, the finite element model used in this study was validated for intact femoral biomechanics [12]; however, the specific construct, including the basicervical fracture pattern, the Gamma nail fixation, and the osteoporotic bone model with multiple lag screw configurations, was not directly validated against cadaveric biomechanical testing or clinical failure patterns. Cadaveric validation studies would be necessary to confirm the reliability of the present computational findings before any consideration of clinical translation. Seventh, a formal sensitivity analysis examining the effects of varying bone density, femoral neck anteversion, fracture-line orientation, fracture gap, implant size, nail angle, lag screw length, friction coefficient, and reduction quality on the biomechanical outcomes was not performed. The consistent directional trends observed across all 12 lag screw configurations within this model suggest internal consistency; however, whether these trends remain robust across a range of patient-specific anatomical and mechanical parameters remains to be determined. Finally, as an in silico study, these findings require further biomechanical and clinical validation. Nevertheless, the present finite element analysis generates a computational hypothesis that may provide a biomechanical rationale for further investigation of lag screw positioning strategies in unstable basicervical IT fractures. Prospective clinical and cadaveric studies are warranted to determine whether the computational trends observed in this study correspond to clinically meaningful differences in fixation failure, cut-out, or postoperative stability in unstable basicervical IT fractures.

## 5. Conclusions

Within the limitations of the present finite element analysis under axial loading conditions, inferior and posterior lag screw positions demonstrated greater biomechanical disadvantage in terms of rotational stability and stress concentration. A relatively superior-to-anterior lag screw position showed the most favorable biomechanical profile in this computational model. These findings are presented as hypothesis-generating computational observations and require further validation through cadaveric biomechanical testing and prospective clinical studies before any consideration of clinical translation.

## Figures and Tables

**Table 1:** Material properties applied for the finite element model analysis.

	Elastic Modulus (E) (MPa)	Poisson’s Ratio (v)
Cortical bone	17,000	0.3
Cancellous bone	445	0.2
Cephalomedullary nail (TI6Al4V)	113,800	0.34

**Table 2:** Results of the rotational change of the proximal fragment in finite element models. All values are expressed in degrees (°), measured using the Bryant angle principle. Increase rates compared with Type 1 are shown in parentheses.

	Varus Collapse (°)	Rotation (°)	Retroversion Collapse (°)
Type 1	5.18	8.42	5.71
Type 2	6.32 (22.0%)	11.87 (41.0%)	6.27 (9.8%)
Type 3	6.94 (34.0%)	11.83 (40.5%)	7.68 (34.5%)
Type 4	5.54 (6.9%)	9.44 (12.1%)	6.06 (6.1%)
Type 5	6.28 (21.2%)	12.3 (46.1%)	6.40 (12.1%)
Type 6	7.26 (40.1%)	12.68 (50.6%)	8.23 (44.1%)
Type 7	6.72 (29.7%)	11.25 (33.6%)	7.32 (28.2%)
Type 8	6.75 (30.3%)	12.66 (50.4%)	7.56 (32.4%)
Type 9	7.54 (45.6%)	13.44 (59.6%)	8.74 (53.0%)
Type 10	6.85 (32.2%)	12.36 (46.8%)	7.55 (32.2%)
Type 11	7.03 (35.7%)	12.92 (53.4%)	7.73 (35.4%)
Type 12	7.85 (51.5%)	13.61 (61.6%)	8.86 (55.2%)

**Table 3:** Results of mean stress over the ROI at the nail construct.

	Mean Stress (MPa)	Increase Rate (%)
Type 1	462	
Type 2	515	11
Type 3	678	47
Type 4	471	2
Type 5	552	19
Type 6	702	52
Type 7	522	13
Type 8	636	38
Type 9	702	52
Type 10	549	19
Type 11	650	41
Type 12	759	64

The yield strength of the nail (Ti6A14V) was 880 MPa.

**Table 4:** Results of mean stress over the ROI at the femoral head.

	Mean Stress (MPa)	Increase Rate (%)
Type 1	4.94	
Type 2	6.58 *	33
Type 3	10.76 *	118
Type 4	5.12 *	4
Type 5	7.16 *	45
Type 6	11.4 *	131
Type 7	6.5 *	33
Type 8	8.67 *	76
Type 9	12.7 *	154
Type 10	7.7 *	56
Type 11	9.3 *	88
Type 12	13.06 *	164

* Higher than the yield strength of osteoporotic cancellous bone (5 MPa).

## Data Availability

The datasets used and/or analyzed during the current study are available from the corresponding author on reasonable request.
